# Application of a protective sleeve is associated with decreased occupational anxiety during endotracheal intubation: a randomized controlled trial

**DOI:** 10.1186/s12871-021-01467-7

**Published:** 2021-10-22

**Authors:** Chaojin Chen, Ning Shen, Liubing Chen, Tongsen Luo, Tianyou Lu, Dezhao Liu, Qi Zhang, Ziqing Hei

**Affiliations:** 1 Department of Anaesthesiology, The Third Affiliated Hospital of Sun Yat-sen University, No. 600 Tianhe Road, Guangzhou, People’s Republic of China; 2 Cell-gene Therapy Translational Medicine Research Center, The Third Affiliated Hospital, Sun Yat-sen University, No.600 Tianhe Road, Guangzhou, People’s Republic of China; 3 Center for Stem Cell Biology and Tissue Engineering, Key Laboratory for Stem Cells and Tissue Engineering, Ministry of Education, Sun Yat-Sen University, Guangzhou, People’s Republic of China; 4 Department of Anaesthesiology, Yuedong Hospital, The Third Affiliated Hospital of Sun Yat-sen University, Meizhou, People’s Republic of China

**Keywords:** Novel protective sleeve, Endotracheal intubation, Anxiety, Anaesthesiologists, Occupational infection, COVID-19

## Abstract

**Background:**

The high risk of cross-infection during tracheal intubation has caused excessive occupational anxiety for anaesthesiologists amid the novel coronavirus disease 2019 (COVID-19) pandemic. Currently, there is no effective way to attenuate their anxiety in clinical practice. We found that anaesthesiologist with better protective equipment might experience decreased levels of anxiety during intubation.

**Methods:**

In this study, 60 patients who underwent intubation and extubation in the operating room were enrolled, and then randomized 1:1 to either wear protective sleeves (protective sleeve group) or not (control group). Visual analogue scale (VAS) was used to measure the anxiety level of anaesthesiologists during intubation. The respiratory droplets of patients on the sleeve, and the anaesthesiologists’ perception including the patient’s oral malodour, exertion, satisfaction degree, waist discomfort and shoulder discomfort were recorded. The patients’ anxiety, oppressed feelings and hypoxia and postoperative complications were all measured and recorded.

**Results:**

Compared with the control group, the anaesthesiologists in protective sleeve group achieved lower anxiety scores and better satisfaction degrees during the process of intubation and extubation (all *P* < 0.05). Respiratory droplets were observed only on the inner side, but not the external side, of the protective sleeves (*P* < 0.001). The incidence of the anaesthesiologists’ perception of patients’ oral malodour was significantly lower in the protective sleeve group (*P* = 0.02) and no patients developed hypoxemia or intubation-related complications in the protective sleeve group.

**Conclusion:**

Using protective devices for intubation might eliminate droplet transmission from patients to anaesthesiologists, while also decreasing their anxiety in a controlled operating room environment.

**Trial registration:**

Chinese Clinical Trial. no. ChiCTR2000030705. Registry at www.chictr.org.cn on 10/03/2020.

**Supplementary Information:**

The online version contains supplementary material available at 10.1186/s12871-021-01467-7.

## Background

The global outbreak of the novel coronavirus disease 2019 (COVID-19) has placed a contagious threat on thousands of health-care workers, especially in situations of asymptomatic infection [1], and personal protective equipment (PPE) shortages [2]. Healthcare workers have been experiencing fear, anxiety, worry, dread and despair under these circumstances where many people are dying [3], and healthcare workers around the world are now experiencing considerable burnout and low job satisfaction during this COVID-19 pandemic [4]. It has been reported that healthcare workers, especially anaesthesiologists, are facing extreme pressures, leading to declining availability and increasing stress [5]. There is an urgent necessity to take measures to curb the potential anxiety and depression among anaesthesiologists.

Excessive occupational anxiety could result from concerns about the risk of cross-infection and the lack of confidence in the safety measures adopted [6, 7]. During intubation, anaesthesiologists need to be very close to patient’s nose and mouth [8] and they are exposed to the aerosols [9]. Moreover, the shortage of PPE has caused thousands of healthcare workers to be infected amid COVID-19 pandemic and exacerbated occupational anxiety [6]. Currently, there is no effective way to attenuate anaesthesiologists’ anxiety in clinical practice.

We recently shared our advice on video laryngoscopy during endotracheal intubation of COVID-19 patients [10], and designed a convenient new device to isolate the patient’s exhaled gas and droplets from the surroundings [11].The anaesthesiologists were asked to use video laryngoscopy and a protective sleeve for intubation amid the COVID-19 outbreak in our hospital. We believe that anaesthesiologists with better protective equipment might experience decreased occupational anxiety during endotracheal intubation, however, few studies have focused on this issue.

Therefore, we conducted a randomized controlled trial to explore the association between the application of the novel protective sleeve and decreased occupational anxiety in anaesthesiologists.

## Methods

### Study design and participants

This was a single centre RCT conducted in the Third Affiliated Hospital of Sun Yat-sen University. The study protocol was approved by the Institutional Review Board of the hospital (approval number: [2020] 02-022-01), and registered with the Chinese Clinical Trial Registry at www.chictr.org.cn on 10/03/2020 (ChiCTR2000030705). This article was prepared following the Consolidated Standards of Reporting Trials (CONSORT) reporting guidelines.

Female and male patients (between 18 and 75 years old) scheduled to undergo endotracheal intubation and extubation for elective surgery in the operating room during March  9^th^ to May 31^st^ 2020, patients with an American Society of Anaesthesiologists class (ASA) I to II, patients with a Mallampati score of I to II, and patients without upper airway abnormalities were included in this study. Patients with anatomical abnormalities of the upper respiratory tract or serious cardiopulmonary diseases, mouth opening < 4 cm, thyromental distance < 6.5 cm, chin-chest distance < 12.5 cm, or BMI > 30 kg.m^−2^ were excluded.

### Sample size

To compare the primary outcome, anxiety of anaesthesiologists during intubation (VAS) between the two groups, we calculated the sample size based on a power of 90% and a 5% type-I error. G*power (downloaded from http://www.gpower.hhu.de/) specified a size of 28 patients per group was required with a Cohen’s d of 0.8 (medium to high effect size), an alpha of 0.05 (one-tailed) and a power of 0.9. Considering potential dropout, the sample size was calculated to be 30 patients per group.

### Randomization

Patients were randomized into either the homemade protective sleeve group (protective sleeve group) or the control group (control group) with a 1:1 allocation ratio, on the basis of a computer-generated randomization number table saved in a sealed envelope. The researchers performing statistical analyses were blinded to the group allocation.

### Study procedures

The protective sleeve has been widely applied in our clinical practice. Written informed consent was obtained from each participant. All of the patients received standard procedure for intubation with or without the protective sleeve, which was carried out by expert anaesthesiologists with a one-to-one relationship. All of the patients were asked to exhale into a Halitosis Detector (TANITA, HC-2126-WH) to measure the oral odour prior to the induction of anaesthesia, using the method previously reported [8]. Both anaesthesiologists and patients reported their baseline anxiety level before entering the operating room on a visual analogue scale (VAS), a line 10 cm in length with which ranges from 0 (no anxiety) to 10 (extreme anxiety), and a higher score indicates more anxiety [12–16].

Before intubation, the endotracheal catheter, laryngoscope blade and mask were respectively connected to their operating holes in the protective sleeve with adhesive tape and surrounded by water sensitive papers (WSPs) that turned from blue to red if in contact with droplets [17, 18]. The mask was securely attached to the protective sleeve through its connection to the ventilator for high-flow oxygen supply and ventilation before anaesthesia (Figure A1). Patients in the protective sleeve group were preoperatively instructed on how to wear the protective sleeve to cover their head and neck when they were awake, so as not to make them feel claustrophobic or increase their anxiety after application of the protective sleeve. The patients were evaluated for their oppressed feelings when wearing the sleeve and for their anxiety score regarding cross-infection related to the possibility of contagious COVID-19 pandemic infection. All anaesthesiologists wore standard medical protective masks, caps and gloves.

The standard anaesthesia protocol consisted of intravenous induction with midazolam (0.1 mg kg^−1^), sufentanil (0.3 mg kg^−1^), propofol (1-2 mg kg^−1^) and cisatracurium (0.2 mg kg^−1^). After induction, the anaesthesiologists exposed the glottis using a nonchanneled UE video laryngoscope (TDC-K series) and performed endotracheal intubation. At the same time, the assistant helped to measure the distance from the patient’s mouth to the operator’s nose (mouth-to-nose distance, MN distance) [8]. After that, the catheter guide wire was removed by an assistant with a disinfectant gauze and the patients were immediately connected to a ventilator. After intubation, ETT was connected to the ventilator and fixed and the protective sleeve was then removed from outside to inside to minimize exposure to any droplets load entrapped in the sleeve. The time to successful intubation was recorded from the moment of picking up the video laryngoscope to the first capnography upstroke after intubation. When the surgery was completed, the patients were covered with a new protective sleeve with a sputum suction tube inside it. The anaesthesiologists conducted endotracheal sputum suction and extubation with the protective sleeve. The anaesthesiologists were asked to report their own anxiety level and their satisfaction degree. The satisfaction degrees were graded as 3 levels: totally satisfied, relatively satisfied and unsatisfied according to earlier report [19]. Of note, if the anaesthesiologists faced a difficult or impossible intubation with the protective sleeve, they were asked to remove the protective sleeve and seek help from a senior anaesthesiologist to conduct routine endotracheal intubation.

In the control group, routine endotracheal intubation and extubation were conducted without a protective sleeve. Patients were asked to breathe calmly whether they wore the protective sleeve or not.

### Data collection and outcomes

Baseline population characteristics, including age, sex, height, weight, BMI, ASA physical status, and Modified Mallampati Score were prospectively collected.

The primary outcome was the anaesthesiologists’ anxiety score about COVID-19 infection during the process of intubation. Secondary outcome variables included the anaesthesiologists’ anxiety score about COVID-19 infection during the process of extubation, perception of the patient’s oral malodour during the process of intubation, the number of respiratory droplets recorded by the water sensitive paper (WSP), anaesthesiologists’s waist and shoulder discomfort and their satisfaction degree, the MN distance, and the lifting strength recorded as previously described [8]. Following intubation, the anaesthesiologists immediately recorded the lifting strength rating, which was defined as the length in centimetres from 0 (no exertion) to 10 (maximal exertion) on the VAS [8]. The patients’ oppressed feelings when wearing the sleeve and anxiety scores were also recorded. In addition, the complications during endotracheal intubation and extubation were also recorded. Waist and shoulder discomfort were judged by the 4-point scale: no, mild, moderate and severe according to our earlier study [8].

### Statistical analysis

All patients meeting the inclusion criteria were analysed and the author who analysed the data was blinded to the grouping of the patients. A one-sample Kolmogorov-Smirnov test was used to test the normality of the continuous data. The quantitative variables, which were normally distributed, were expressed as the mean ± SD, and analysed using Student’s t-test. Nonnormally distributed data, which included the anxiety score, age, intubation time and MN distance, were expressed as the median (interquartile range), and analysed by Mann-Whitney U-test. Spearman’s rank correlation test was used to analyse the association. Qualitative data are presented as frequencies with percentages, and were analysed by the Pearson χ^2^ test or Fisher’s exact test. Differences were considered significant when a two-sided *P* value was less than 0.05. All data analysis was performed using SPSS for Windows V.16.0 (SPSS Inc., Chicago, Illinois, USA). The researchers that performed the statistical analyses were blinded to the group allocation.

## Results

**Fig. 1:**
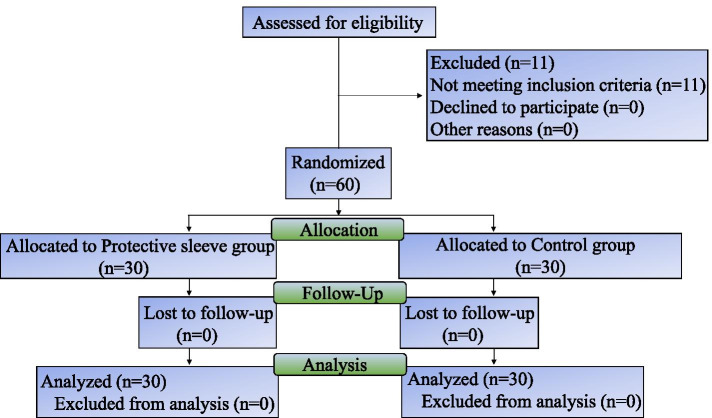
Flow diagram of the process through the phases of the trial

A total of 71 patients undergoing elective surgery were assessed for eligibility and 60 of them met the inclusion criteria and completed the trial. The final analysis included 30 patients with protective sleeves (protective sleeve group) and 30 patients without protective sleeves (control group) during intubation and extubation (Fig. 1). The baseline and clinical characteristics of both groups did not significantly differ with regard to age, sex, height, weight, BMI, ASA physical status, modified Mallampati score or oral malodour score (Table 1).

**Table 1:** Baseline characteristics of participants between the two groups

Variables	Protective sleeve group (***n*** = 30)	Control group (***n*** = 30)	***P -*** value
Sex, n (%)			0.432
Male	11 (36.67)	14 (46.67)	
Female	19 (63.33)	16 (53.33)	
Age, y	45 (34, 54)	42 (29.75,56.25)	0.492
Height, cm	161.57 ± 8.52	163.77 ± 9.19	0.34
Weight, kg	60.22 ± 9.58	61.43 ± 12.86	0.679
BMI (kg m^−2^), n (%)			0.264
< 18.5	1 (3.33)	4 (13.33)	
18.5 to 23.9	15 (50.00)	17 (56.67)	
24 to 28	13 (43.33)	7 (23.33)	
> 28	1 (3.33)	2 (6.67)	
ASA classification, n (%)			0.438
I	13 (43.33)	16 (53.33)	
II	17 (56.67)	14 (46.67)	
Modified Mallampati Score, n (%)			1.000
I	22 (73.33)	22 (73.33)	
II	8 (26.67)	8 (26.67)	

Abbreviations: *ASA* American Society of anaesthesiologists, *BMI* body mass index

Values are mean ± SD, median (inter-quartile range) or n (%), *P-value < 0.05

The baseline anxiety scores of the anaesthesiologists and patients did not differ between the protective sleeve and control groups (both *P* > 0.05; Table 2). However, the anaesthesiologists’ anxiety scores were significantly lower in the Protective sleeve group compared with those in the Control group during intubation and extubation (both *P* < 0.001; Table 2). Similarly, the anxiety scores of the patients were significantly lower when wearing the protective sleeve (*P* < 0.05; Table 2).

**Table 2:** The anaesthesiologists and patients’ anxiety scores

Variables	Protective sleeve group(***n*** = 30)	Control group (***n*** = 30)	***P-*** value
Operator’s baseline anxiety score, n (%)			1.000
0 to 3	28 (93.33)	28 (93.33)	
4 to 6	2(6.67)	2 (6.67)	
7 to 10	0 (0)	0 (0)	
Operators’ anxiety score during intubation, n (%)			0.001*
0 to 3	29 (96.67)	18 (60.00)	
4 to 6	1 (3.33)	6 (20.00)	
7 to 10	0 (0)	6 (20.00)	
Operators’ anxiety score during extubation, n (%)			0.001*
0 to 3	29 (96.67)	18 (60.00)	
4 to 6	1 (3.33)	4 (13.33)	
7 to 10	0 (0)	8 (26.67)	
Patient’s baseline anxiety score, n (%)			0.936
0 to 3	23 (76.67)	24 (80.00)	
4 to 6	5 (16.67)	4 (13.33)	
7 to 10	2 (6.67)	2 (6.67)	
Patient’s anxiety score before intubation, n (%)			0.014*
0 to 3	28 (93.33)	19 (63.33)	
4 to 6	1 (3.33)	6 (20.00)	
7 to 10	1 (3.33)	5 (16.67)	

Abbreviations: *WSP* water sensitive paper, *IQR* interquartile range

The anxiety about being infected by COVID-19 was defined according to VAS: from 0 (no anxiety) to 10 (extreme anxiety); Values are mean ± SD, median (inter-quartile range) or n (%), *P-value < 0.05

To confirm the isolating effect of the protective sleeve, we used the WSP to record the patients’ respiratory droplets. The number of droplets recorded by the WSP pasted on the inner side of the protective sleeve was 3 (2,5) and 3.5 (1,5) for intubation and extubation, respectively. However, no droplets were recorded on the external WSP side of the protective sleeve (Table A1). In addition, we also measured the anaesthesiologists’ perception of halitosis during intubation to reflect the sleeve’s isolating effect. The baseline oral malodour score did not differ between the two groups prior to the induction of anaesthesia (*P* = 0.704; Table 3). However, the incidence of the anaesthesiologists’ perception of halitosis in patients during intubation was significantly lower in the protective sleeve group than in the control group (0% vs. 16.67%, *P* = 0.02; Table 3).

**Table 3:** Secondary endpoints of the study

Variables	Protective sleeve group (***n*** = 30)	Control group (***n*** = 30)	***P-*** value
Patients’ oppressed feeling	0 (0)	0 (0)	1.000
Baseline oral odour score, n (%)			0.704
0 to 2	27(90.00)	25(83.33)	
3 to 5	3(10.00)	5(16.67)	
Perception of patients’ oral malodour	0 (0)	5 (16.67)	0.02*
Intubation time(s)	93 (75.75,125.50)	54 (41,83)	< 0.001*
MN distance (cm)	33 (30, 38.25)	36.5 (30.75,42)	0.122
Lifting strength scale, n (%)			0.182
0 to 3	23 (76.67)	16 (53.33)	
4 to 6	4 (13.33)	10 (33.33)	
7 to 10	3 (10.00)	4 (13.33)	
Waist discomfort, n (%)			1.000
No	28 (93.33)	27 (90.00)	
Mild	2 (6.67)	3 (10.00)	
Moderate	0 (0)	0 (0)	
Severe	0 (0)	0 (0)	
Shoulder discomfort, n (%)			0.237
No	30 (100)	27 (90.00)	
Mild	0 (0)	3 (10.00)	
Moderate	0 (0)	0 (0)	
Severe	0 (0)	0 (0)	
Satisfaction degree of operators, n (%)			0.016*
Totally satisfied	8 (26.67)	1 (3.33)	
Relatively satisfied	22 (13.33)	28 (30.00)	
Unsatisfied	0(0)	1(3.33)	

Lifting strength was assessed by VAS: from 0 (no exertion) to 10 (maximal exertion)

Waist and shoulder discomfort were judged by the 4-point scale: No, Mild, Moderate and Severe

Satisfaction degree was graded as Totally satisfied, Relatively satisfied and Unsatisfied

Values are mean ± SD, median (inter-quartile range) or n (%), *P-value < 0.05

To evaluate the ease of use of the protective sleeve, we also measured the exertion and waist discomfort and shoulder discomfort of the anaesthesiologists. Their exertion was comparable in the protective sleeve group and control group (*P* = 0.182; Table 3) and there was no significant difference between the two groups with regard to waist discomfort and shoulder discomfort (both *P* > 0.05; Table 3).The result was consistent with our previous study [8]. However, the control group had a remarkably decreased overall satisfaction degree compared with the protective sleeve group (*P* = 0.016; Table 3).

To evaluate the safety of the protective sleeve, we recorded the intubation time and the patients’ oppressed feelings and hypoxia during the study. We found that the intubation time in the experimental group was longer than that in the control group [93 (75.75,125.50) vs. 54 (41,83), *P* < 0.001; Table 3]. No patient felt oppressed when wearing the protective sleeve before induction and no patient in the study developed hypoxia (SpO_2_ < 95%) during intubation. There was no significant difference in MN distance between the two groups (*P* > 0.05; Table 3). Moreover, there was no significant correlation between the anaesthesiologists’ height and the MN distance (Figure A2).

## Discussion

In a time of uncertainty, fear, and real dangers for health-care workers [20, 21], the current study showed that the use of protective devices for intubation and extubation might eliminate droplet transmission from patients to anaesthesiologists performing intubation, while also decreasing their anxiety and not adversely affecting patients’ safety in a controlled operating room environment.

Notably, the anxiety of anaesthesiologists is caused by a variety of factors, including exposure risk, limited PPE, long working hours, and the overall uncertainty surrounding COVID-1 [22]. It has been suggested that to prevent occupational stress, instead of increasing economic or other rewards, it is more important to reorganize the work, to reduce the effort made by each anaesthesiologist [22]. However, excessive number of patients and relatively insufficient number of anaesthesiologists have resulted in a high workload for Chinese anaesthesiologists [23]. In this study, we aimed to explore the increased psychological stress about the cross-infection challenges during intubation [24]. Our results confirmed that improving the anaesthesiologists’ PPE amid the COVID-19 pandemic may be beneficial for occupational stress relief. Moreover, we also measured the patients’ anxiety score before anesthesia induction in the study, as we thought that the patients were also at risk of COVID-19 cross-infection from the anesthesiologists who might have an asymptomatic COVID-19 infection. Our results showed that application of the protective sleeve help to calm not only the anesthesiologists but also the patients. To the best of our knowledge, this is the first study to show that the application of protective devices for intubation is associated with decreased anxiety of anaesthesiologists and patients.

These results may not come as a surprise to many anaesthesiologists who are used to using protective sleeves for intubation and extubation [25, 26]. Different from other intubation boxes and sheets that have reported efficacy data limited to simulated conditions [26–29], our study is unique in its enrolment of actual patients undergoing endotracheal intubation in an operating room environment. This study based on our earlier work further confirmed that the novel protective sleeve was able to create a relatively isolated environment during intubation and extubation, as reflected by the number of droplets recorded by the WSP, and the perception of the patient’s oral malodour. Although many studies have proven that they could not block aerosols completely [28, 30], and that aerosols were contaminated in their inner surface, which may cause exposure during removal of the device and stylet, we think that main purpose of devices designed by anaesthesiologists around the world is not to isolate aerosols completely, but to strengthen protection during tracheal intubation, and intubation boxes and sheets cannot replace the positive pressure protective clothing. During the COVID-19 pandemic, although anaesthesiologists need to conduct tracheal intubation every day, they are not able to wear protective clothing and screens all of the time. This extra layer of protection further reduced the direct contact between anaesthesiologists and patients during intubation and reduced the anxiety of the anaesthesiologists.

The safety of this new device is concern, especially in critical situations. The setup of this protective sleeve requires time and hypoxemic patients may be at risk of desaturation. In our study, none of the patients developed hypoxemia or other complications during intubation, and the protective sleeve did not cause any oppressed feelings in patients, and it did not affect the anaesthesiologists’ operations, as reflected by comparable exertion, waist and shoulder discomfort. However, due to the limited space for using a stylet in the protective sleeve, the average intubation time was slightly longer than that of the control group, which is consistent with an earlier report [31–33]. Moreover, there is a real possibility of accidental tube displacement during the removal of the sleeve. The intubation time was clinically acceptable, and no patient experienced hypoxemia or accidental tube displacement in our study; furthermore, we found that experienced anaesthesiologists could perform intubation using a protective sleeve with minimally increased time and they could fluently remove the sleeve, thereby indicating that sufficient training on using these protective devices is necessary in clinical practice.

Limitations of this study should be considered. First, neither patients nor anaesthesiologists were blinded to the grouping, since the anaesthesiologists who were used to using the sleeve were likely biased to believe it was effective, and suffered greater anxiety and dissatisfaction when they did not have access to it. Thus, the results might involve bias and placebo effects and need further confirmation. Second, although we informed patients and anaesthesiologists in the preoperative interviews that we only assessed anxiety about COVID-19 cross-infection, the preoperative anxiety of patients and anaesthesiologists has many components, such as the degree of surgical difficulty and the time of the day; these confounding factors might affect the results. We did not record and compare the other factors associated with anxiety, and it is difficult to rule out all of these components completely. Third, although this device makes anaesthesiologists feel safer, and increases their satisfaction degree during intubation, it probably also reduces clinician vigilance and causes them to slightly alter their behavior in other respects to subconsciously accept more risk, which is called “risk compensation”. It is unlikely that this behavior change would be seen in this trial due to it being observed (the Hawthorn effect), but it would likely occur in later use. Therefore, this aspect should be emphasized when training anaesthesiologists in using the protective sleeves. Fourth, the study was a single-centre RCT with a relatively small sample size. Therefore, these results require further confirmation in large-scale multicentre studies. Fifth, the protective equipment might increase the cost of patients amid the COVID-19 pandemic. Sixth, this is not a psychological assessment study, and this is a clinical anesthesiologists satisfaction study. Therefore, we did not intend to thoroughly assess each anesthesiologist’s psychological status before this clinical study, and we prefer to evaluate only their satisfaction.

## Conclusion

Using protective devices for intubation might eliminate droplet transmission from patients to anaesthesiologists, while also decreasing their anxiety in a controlled operating room environment. The results of this study indicated that anaesthesiologists with better PPE might have more confidence and experience decreased anxiety during intubation.

## Supplementary Information

**Additional file 1.**

## Data Availability

The results in the study could be accessed via the corresponding author upon reasonable request.

## Abbreviations

COVID-19: Coronavirus disease
PPE: Personal protective equipment
ASA: American Society of Anaesthesiologists class
VAS: Visual analogue scale
MN: Mouth to nose
