# Supplementary material for: Application of a protective sleeve is associated with decreased occupational anxiety during endotracheal intubation: a randomized controlled trial
Source: BMC Anesthesiol. 2021 Oct 22;21:251. doi: 10.1186/s12871-021-01467-7 (PMC8532101; doi:10.1186/s12871-021-01467-7)
Supplement: Supplementary file 1 — Additional file 1. [file 12871_2021_1467_MOESM1_ESM.docx]

**Additional file**

**Application of a Protective Sleeve** **is Associated with Decreased Occupational Anxiety During** **Endotracheal Intubation: a Randomized Controlled Trial**

Chaojin Chen^1,2,3#^; Ning Shen^1#^; Liubing Chen^1#^; Tongsen Luo^1^; Tianyou Lu^1^; Dezhao Liu^1^; Qi Zhang^2, 3^; Ziqing Hei^1,4*^

^1^Department of Anesthesiology, The Third Affiliated Hospital of Sun Yat-sen University, Guangzhou, People’s Republic of China.

^2^Cell-gene Therapy Translational Medicine Research Center, The Third Affiliated Hospital, Sun Yat-sen University, Guangzhou, People’s Republic of China

^3^Center for Stem Cell Biology and Tissue Engineering, Key Laboratory for Stem Cells and Tissue Engineering, Ministry of Education, Sun Yat-Sen University, Guangzhou, Guangzhou, People’s Republic of China

^4^Department of Anesthesiology, Yuedong Hospital, The Third Affiliated Hospital of Sun Yat-sen University, Meizhou, People’s Republic of China.

**Table A1. Number of droplets recorded by the WPS pasted on protective sleeve during intubation and extubation.**

| **Variables** | **Outer-side (n=30)** | **Inner-side (n=30)** | ***P-* value** |
| --- | --- | --- | --- |
| Intubation |  |  |  |
| Number of droplets per sleeve | 0 (0, 0) | 3 (2, 5) | 0.000* |
| Number of sleeves with droplets >0 | 0 (0) | 26 (86.67) | 0.000* |
| Extubation |  |  |  |
| Number of droplets per sleeve | 0 (0) | 3.5 (1,5) | 0.000* |
| Number of sleeves with droplets >0 | 0 (0) | 26 (86.67) | 0.000* |

Values are median (inter-quartile range) or n (%), **P*-value<0.05.

**Figure A1. Using the protective sleeve as PPE during endotracheal intubation.**

The figure was created by the first author, Dr. Chaojin Chen. We confirm that it has not been used in any other published article. We have the full permissions to use this image. ETT, endotracheal tube; Blade, laryngeal blade.


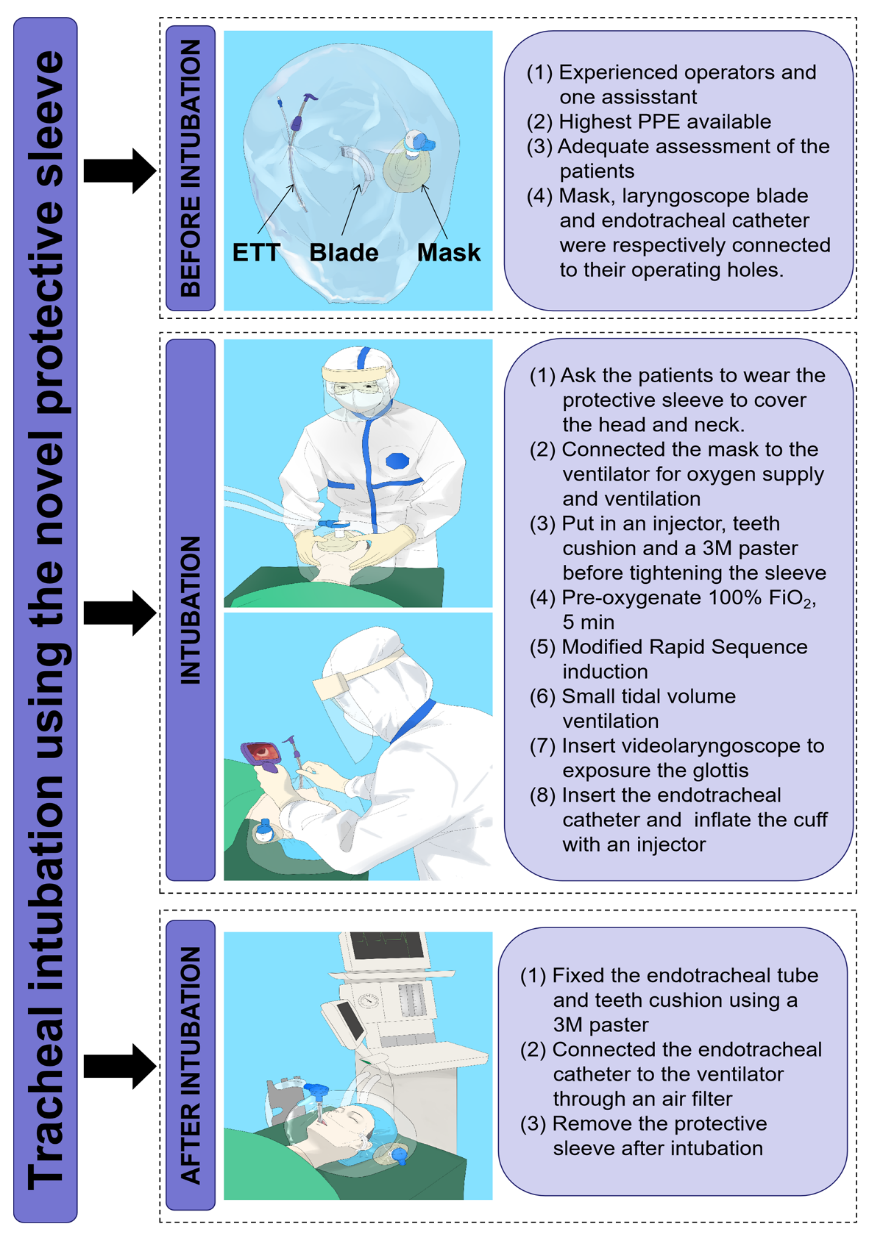


**Figure A2 Correlation analysis between MN distance and Height.**


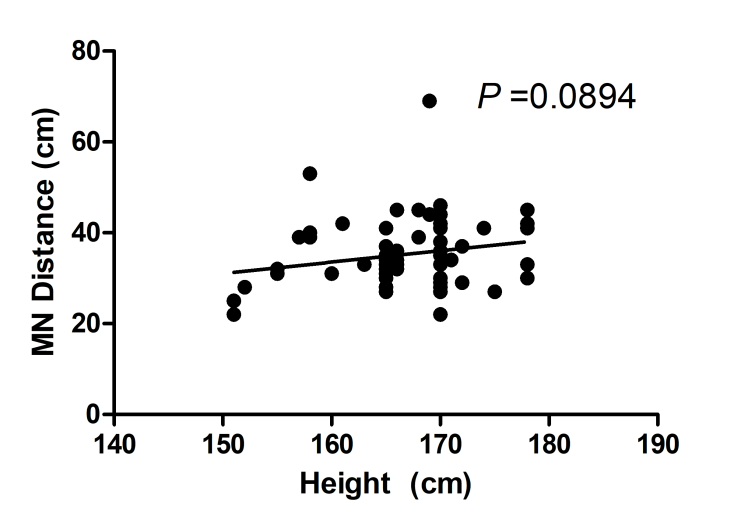


MN distance: distance from patient’ mouth to anesthetist’s nose when intubation.
